# Optically transparent vertical silicon nanowire arrays for live-cell imaging

**DOI:** 10.1186/s12951-021-00795-7

**Published:** 2021-02-17

**Authors:** Roey Elnathan, Andrew W. Holle, Jennifer Young, Marina A. George, Omri Heifler, Andriy Goychuk, Erwin Frey, Ralf Kemkemer, Joachim P. Spatz, Alon Kosloff, Fernando Patolsky, Nicolas H. Voelcker

**Affiliations:** 1 Faculty of Pharmacy and Pharmaceutical Sciences, Monash University, Parkville, Vic 3052 Australia; 2 Department of Materials Science and Engineering, Monash University, 22 Alliance Lane, Clayton, Vic 3168 Australia; 3 Melbourne Centre for Nanofabrication, Victorian Node of the Australian National Fabrication Facility, Victoria, Australia; 4 Mechanobiology Institute, National University of Singapore, Singapore, Republic of Singapore; 5 Department of Biomedical Engineering, National University of Singapore, Singapore, Republic of Singapore; 6 School of Chemistry, The Raymond and Beverly Sackler Faculty of Exact Sciences, Tel-Aviv University, Tel Aviv, Israel; 7 The Center for Nanoscience and Nanotechnology, Tel-Aviv University, 69978 Tel Aviv, Israel; 8 Arnold Sommerfeld Center for Theoretical Physics and Center for NanoScience, Department of Physics, Ludwig-Maximilians-Universität München, 80333 Munich, Germany; 9 INM-Leibnitz Institute for New Materials, Campus D2 2, 66123 Saarbrücken, Germany; 10 Department of Cellular Biophysics, Max Planck Institute for Medical Research, 69120 Heidelberg, Germany; 11 Department of Applied Chemistry, Reutlingen University, 72762 Reutlingen, Germany; 12 Department of Biophysical Chemistry, University of Heidelberg, 69120 Heidelberg, Germany

**Keywords:** Nanowires, Cell–material interface, Live-cell phase-contrast imaging, Silicon, Glass substrate

## Abstract

Programmable nano-bio interfaces driven by tuneable vertically configured nanostructures have recently emerged as a powerful tool for cellular manipulations and interrogations. Such interfaces have strong potential for ground-breaking advances, particularly in cellular nanobiotechnology and mechanobiology. However, the opaque nature of many nanostructured surfaces makes non-destructive, live-cell characterization of cellular behavior on vertically aligned nanostructures challenging to observe. Here, a new nanofabrication route is proposed that enables harvesting of vertically aligned silicon (Si) nanowires and their subsequent transfer onto an optically transparent substrate, with high efficiency and without artefacts. We demonstrate the potential of this route for efficient live-cell phase contrast imaging and subsequent characterization of cells growing on vertically aligned Si nanowires. This approach provides the first opportunity to understand dynamic cellular responses to a cell-nanowire interface, and thus has the potential to inform the design of future nanoscale cellular manipulation technologies. 
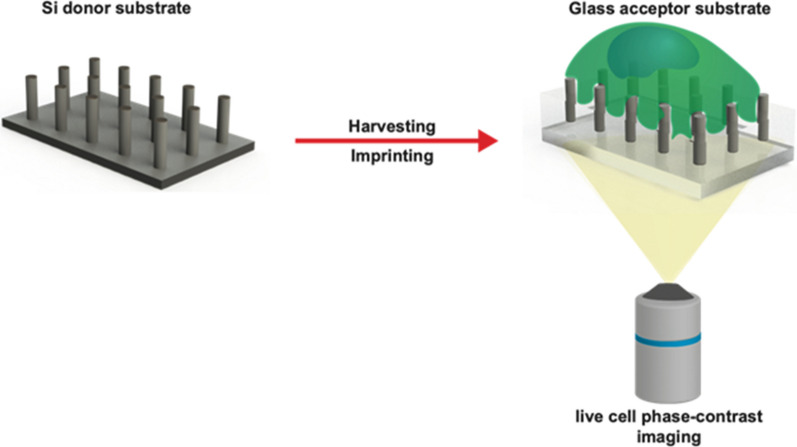

Significant multidisciplinary progress in cellular nanotechnology has led to engineered nano-bio cellular interfaces that can stimulate and leverage cellular processes at the nanoscale [1–5]. Well-defined nanomaterial morphologies—in particular, vertically-aligned nanostructures such as nanowires, nanostraws, and nanotubes (NW, NS, and NT)—can now be interfaced with cellular systems to manipulate and interrogate cell function, behaviour, and fate [6–15]. Despite these advances, the biological response to engineered nano-bio cellular interfaces remains poorly understood. Thus, the development of new tools for directly probing this response is likely to lead to the development of novel fundamental research applications and ex vivo cell-based therapies [16–19].

The ability of these nanostructures to elicit functional cellular responses at the cell–material interface—such as intracellular delivery, biomolecular extraction (nanobiopsy), nanoelectrode-based electrophysiology, biosensing, and mechanotransduction—arises from their salient advantages in multiple independent parameters: geometric/architectural flexibility, minimal invasiveness, and the ability to simultaneously interface with large numbers of cells [20–25].

Despite implementation of these platforms in a variety of advanced cellular applications—such as in vivo and ex vivo gene editing, recording cellular action potential, and immunomodulation—the development of this burgeoning field is hindered by a lack of tools allowing for direct, rapid, and dynamic visualization of living cells interacting with these nanostructures [26–32].

Recent advances in combinatorial nanofabrication routes now offer precise control over SiNWs growth, topological parameters, and array architecture, overcoming the limitations of conventional fabrication that have restricted cell–NW studies [33–35]. Combining colloidal lithography techniques, such as convective assembly and self-assembly at liquid–liquid interfaces, with either metal-assisted chemical etching (MACE) or deep reactive ion etching (DRIE), enables controlled VA-SiNWs growth [36–38]. Other recent nanoscale patterning paradigms for overcoming bottlenecks in fabricating fully tunable SiNW arrays over large areas have been demonstrated by creating defined sequential binary assemblies at fluid interfaces (combining two types of microgel sizes), and transferring them onto a Si substrate to generate a lithographic etching mask to grow binary VA-SiNW arrays [39].

Transfer of vertically and horizontally configured nanostructures from one substrate to another has been demonstrated via nanotransplantation printing [40]. Although this has mostly been applied to horizontally aligned in-plane nanostructures, it has also been utilized for the transfer of VA-SiNWs into flexible PDMS substrates for multispectral imaging applications [41–45].

These developments in nanofabrication routes have opened the doors for significant interdisciplinary opportunities. Programming SiNW arrays with adaptable architectures to function as nanoscale-enhanced tools allows for the direct and diverse manipulation of large cell populations [46–50]. Despite this, there has been almost no progress toward live-cell characterization of cell–nanostructure interfacial interactions. To address this, an efficient, flexible, and non-destructive nanofabrication route that is compatible with optical microscopy is needed.

Visualizing cells on Si nanostructures mostly requires fixation and subsequent epifluorescence microscopy due to the opaque nature of Si substrates. Fixed cells can also be imaged at higher resolution with the aid of advanced super-resolution microscopies, including stimulated emission depletion microscopy, stochastic optical reconstruction microscopy, and combinations of these modalities [51, 52]. Additional imaging modalities, including transmission electron microscopy (TEM), focused ion beam scanning electron microscopy (FIB-SEM), and electron tomography have all been highly effective for imaging fixed cells at the nanoscale cell–material interface [53, 54]. Specifically, FIB-SEM lift-out of cell-nanomaterial composites—generated via FIB milling and placed directly in a customized TEM grid—in conjunction with the development of new ultrathin sectioning methods have led to a deeper understanding of how surface-based nanostructures can affect intracellular processes [55]. While these imaging strategies have greatly advanced our understanding of the cell-material interface, none are compatible with dynamic imaging of live cells on nanostructured surfaces.

## Results and discussion

Here, we present a novel experimental approach that enables transfer of vertically-aligned Si nanowires from their original Si substrate onto an optically transparent glass substrate while preserving the geometrical features. We demonstrate the potential of these substrates for label-free live-cell phase contrast imaging of dynamic cellular processes such as cell division, morphology change, and migration. This approach will advance our understanding of cellular responses to extracellular bio-physical cues—which in turn is likely to improve the design of future cellular manipulation technologies.

**Fig. 1:**
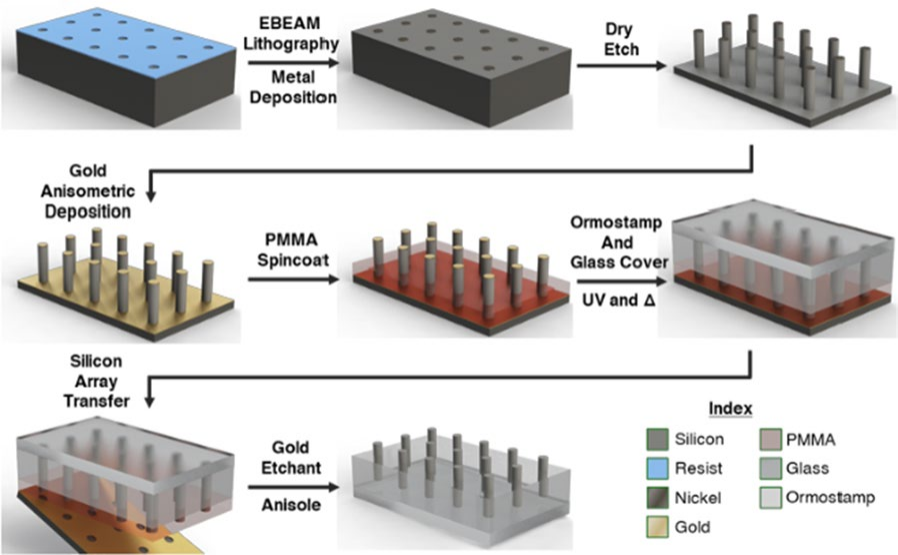
Steps from the fabrication of programmed vertically configured SiNW arrays, and their post-detachment from the Si substrate and their subsequent printing onto the glass substrate

**Fig. 2:**
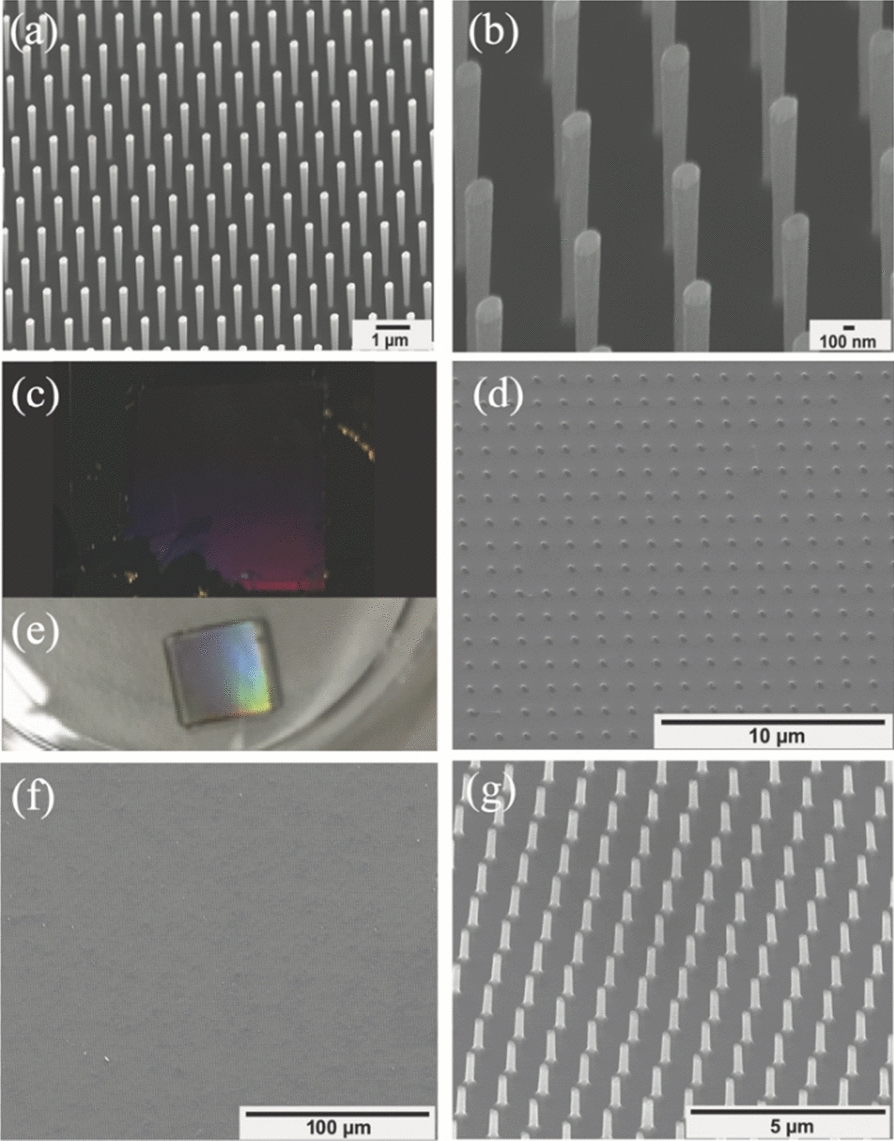
SiNW fabrication and their subsequent transfer onto an optically transparent substrate. **a**, **b** SEM images of the negatively tapered SiNW arrays fabricated via e-beam lithography and DRIE, (**a**) the zoom-out, and (**b**) the zoom-in. **c**, **d** Optical and SEM showing the Si donor substrate post-harvest. **e**–**g** Optical image showing SiNW transfer onto a glass substrate (**e**); and tilt SEM images of a zoom-out and zoom-in of the transferred SiNW array onto the glass substrate

Figure 1 depicts in detail the nanofabrication pipeline for the SiNW array harvesting and subsequent transfer onto transparent glass substrates. First, e-beam lithography was used to directly write a customized lithographical mask array of nanoscale-circles—200 nm diameter, 1 µm pitch—on an electron-resist film coated onto a Si substrate. Second, a 50 nm thick Ni layer was evaporated and the resist layer lifted off, resulting in the formation of Ni disk-like shapes that served as programmable lithographical etching masks. Third, SiNW arrays were generated using DRIE, and the Ni etch-masks removed. This step was followed by deposition of a 30 nm thick Au-layer over the NW array using e-beam direct-angle evaporation. Fourth, a PMMA layer was deposited by spin coating, followed by immersion in an Au-etchant solution. This step is crucial for selectively removing the residual Au-layer on the top and sidewalls of the NWs. The remaining Au layer on the bottom of the SiNW substrate served as an anti-adhesion agent, which later promoted the NW arrays’ selective harvesting and their subsequent transfer from the Si donor substrate onto the acceptor transparent glass substrate. The PMMA resist layer served as a lower embedding layer that could be further removed after the imprint process. Fifth, a silicate-based UV-curable Ormostamp solution was cast on top of the donor substrate NW array; the acceptor glass substrate was then placed precisely above the SiNW arrays (sandwich-type interface), followed by a UV curing step. Sixth, once cured, the Si-donor and glass-acceptor substrates were mechanically separated by placing a razor blade between the samples (only at the corner, without touching the pillar array), while applying a small force.

(Figure 2a, b) shows zoom-out and zoom-in SEM images SiNW arrays that were generated using DRIE. (Figure 2c, d) shows optical and SEM images of the Si-donor substrate after SiNW array harvesting. The NW array was successfully transferred onto the glass substrate along with its embedded cured-Ormostamp/PMMA/Au layers. The NW array was exposed through immersion in Au-etchant solution followed by removal of the top PMMA layer via immersion in anisole solution. The lower layer of the exposed nanowires remained embedded in the optically-transparent Ormostamp. This process results in accurate, precise, and high-efficiency SiNW array transfer from Si to glass, preserving the original programmable design (Fig. 2e-g; optical and SEM images). (Additional file 1: Figure S2) shows additional Si geometry onto an optically transparent substrate. (Additional file 1: Figure S3) shows incomplete NW transfer arrays onto the glass substrates, while (Additional file 1: Figure S4) shows destructive NW transfer on the glass substrates.

**Fig. 3:**
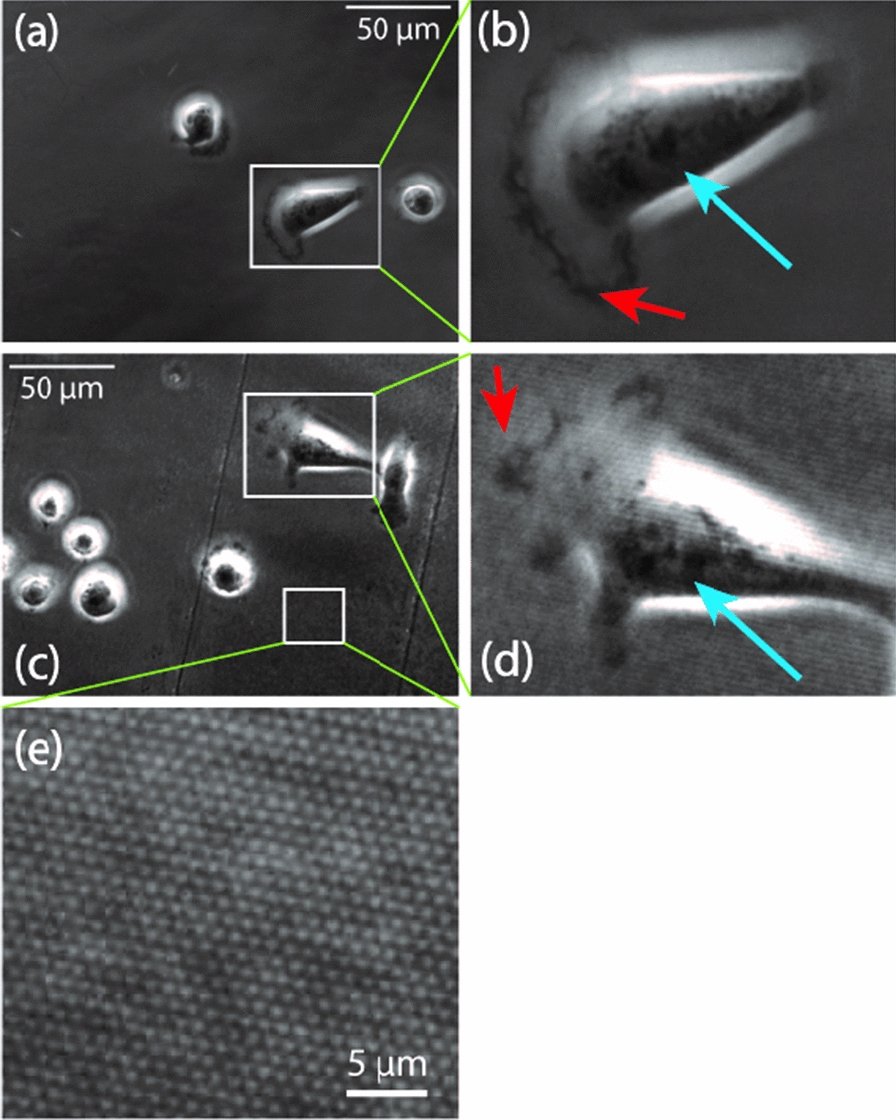
Phase contrast images of MDA-MB-231 breast cancer cells cultured for over 3 h on flat glass (**a**, **b**) or transparent SiNW surfaces (**c**, **d**). Insets (**b**, **d**) illustrate the label-free localization of cellular nuclei (blue arrows) and lamellipodia (red arrows). Inset (**e**) illustrates the direct visualization of nanowire arrays by means of phase contrast microscopy

Arrays of SiNWs on glass substrates allows for the dynamic observation of cell behavior as a function of nanowire interaction over long time scales, (Fig. 3, Additional files 2, 3: Movies 1, Movie 2). Using phase contrast microscopy, cellular features like the nucleus (Fig. 3b, d; blue arrows) and lamellipodia (Fig. 3b, d; red arrows) can be observed in living MDA-MB-231 breast cancer cells growing on flat control (Fig. 3a) or SiNW substrates (Fig. 3c).

**Fig. 4:**
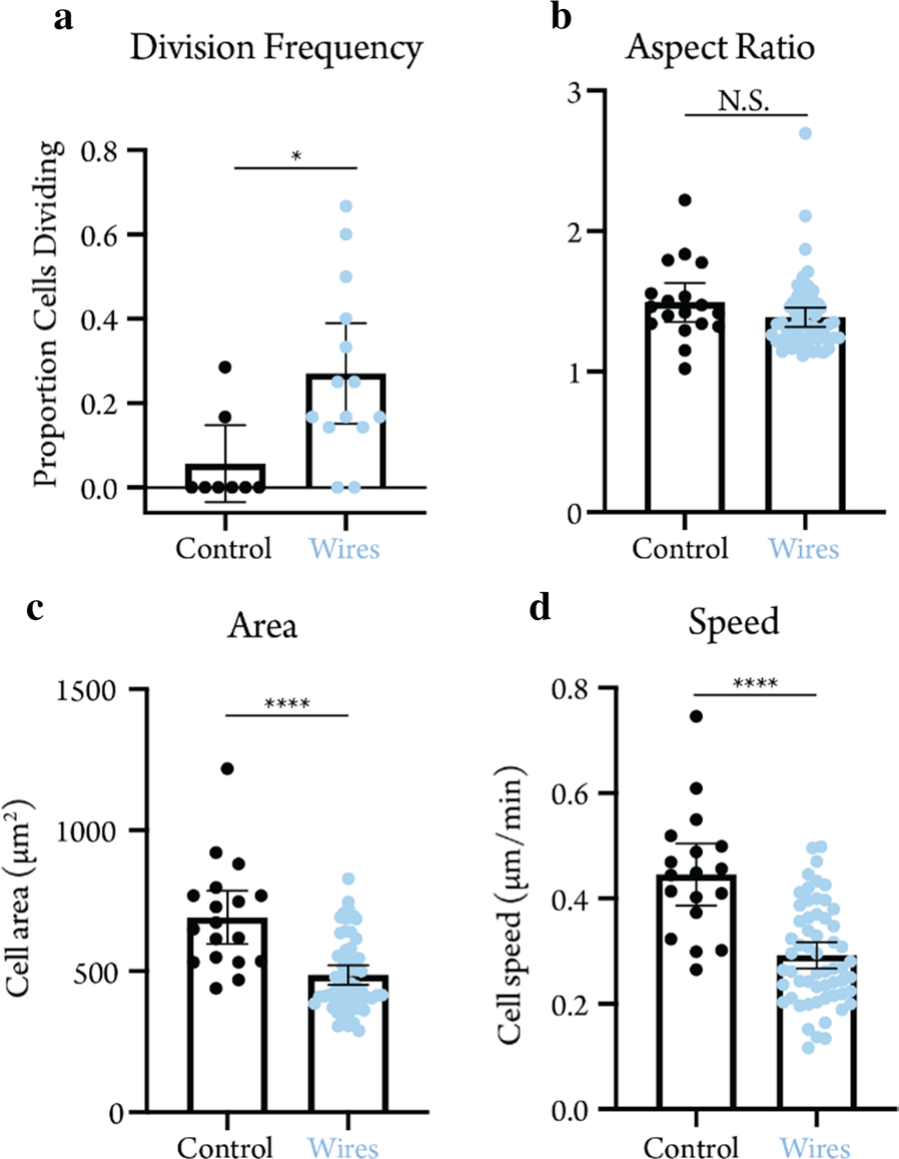
Cell behaviour as a function of SiNW substrate. For division frequency (**a**), each circle corresponds to the proportion of cells dividing in a single field of view. For all other plots (**b**–**d**), each circle corresponds to a single cell averaged over 24 h. Error bars represent 95% confidence intervals of the mean. *p < 0.05

As time lapse microscopy generates hundreds of label-free phase contrast images, the manual segmentation of individual cells is challenging. To address this, we trained a convolutional neural network to automatically identify and track cell outlines (Additional file 4: Movie 3). To leverage the unique advantages of our transparent SiNW arrays, we focused on dynamic cellular characteristics that would be difficult or impossible to observe in fixed cells. While fully spread cells were observed on SiNW substrates (Fig. 3d), we found that the presence of SiNWs results in significant decreases in cell area and cell velocity of MDA-MB-231 cells (Fig. 4c, d), although the initial cell viability, as measured by the number of attached cells per mm [2], was not affected (Figure S5). Interestingly, the number of cells undergoing division events on SiNW surfaces was significantly higher (p = 0.014) than on control glass substrates, (Fig. 4a).

This increased division frequency, which causes cells to round up, likely also accounts for the slight decrease in cellular aspect ratio on SiNW surfaces (Fig. 4b). To date, nanowire structures have not been implicated in regulating the cell cycle, which highlights the unique void these transparent structures fill in the field of nanoscale material–cell interfaces. Future studies will likely leverage this ability to probe these phenomena, as well as other dynamic cell behaviours, like cell spreading, contact guidance, and collective migration. Given that the highly metastatic MDA-MB-231 cells used for this experiment feature a relatively fast doubling time, it is possible that the effect on division frequency may be tempered in other lines that divide less frequently. Perhaps most intriguingly, the ability to visualize the nanowire structures directly via phase contrast microscopy (Fig. 3e) hints at the possibility of correlating directional cell behaviour, either at the whole cell scale area with respect to individual protrusions, to the alignment of the long-range nanowire patterns.

This novel experimental approach enables harvesting of vertically configured SiNWs from a Si-donor substrate and their subsequent transfer onto a glass substrate with minimal artifacts. A considerable advantage of this approach, apart from high efficiency harvesting and subsequent transfer onto an optically transparent substrate, is compatibility with live-cell phase contrast imaging. We have demonstrated proof-of-concept of dynamic characterization of cellular morphology, division, and migration, without the need for immunocytochemical markers, on a nanowire-structured substrate. This approach opens a new dimension for the ongoing study of cellular responses to nanoscale extracellular biophysical cues, which is likely to facilitate the development of improved nanoscale cellular manipulation technologies.

## Supplementary Information

**Additional file 1: Fig. S1.** Glass substrate following the application of Ormostamp layer and separation from the Si substrate. The transferred Si-NW array with its bottom Au coating is indicated by the arrows. **Fig. S2.** Zoom-out and zoom-in SEM images showing an additional imprinted NW geometry: height 1 µm and 0.8 µm spacing. **Fig. S3.** SEM images showing incomplete NW transfer array onto the glass substrate. Typically, yielding % failure <10%. **Fig. S4.** SEM images showing destructive NW transfer array on the glass substrate. Typically, yielding % failure <10%. **Fig. S5.** Nanowire surfaces do not significantly affect initial cell viability less than 3 hours after plating.

**Additional file 2: Video 1.** Phase contrast video of MDA-MB-231 cells on a flat glass substrate.

**Additional file 3: Video 2**. Phase contrast video of MDA-MB-231 cells on a transparent VA-SiNW substrate.

**Additional file 4: Video 3**. Video of MDA-MB-231 cells with overlaid automatic cell segmentation results (p (x,y)) and cell contours at p (x,y) = 0.25. The glyph 'x' denotes the respective centroid, with the current object ID.

## Data Availability

All data generated or analysed during this study are included in this published article [and its Additional files 1, 2, 3,4].
