# Supplementary material for: Optically transparent vertical silicon nanowire arrays for live-cell imaging
Source: J Nanobiotechnology. 2021 Feb 17;19:51. doi: 10.1186/s12951-021-00795-7 (PMC7890818; doi:10.1186/s12951-021-00795-7)
Supplement: Supplementary file 1 — Additional file 1: Fig. S1. Glass substrate following the application of Ormostamp layer and separation from the Si substrate. The transferred Si-NW array with its bottom Au coating is indicated by the arrows. Fig. S2. Zoom-out and zoom-in SEM images showing an additional imprinted NW geometry: height 1 µm and 0.8 µm spacing. Fig. S3. SEM images showing incomplete NW transfer array onto the glass substrate. Typically, yielding % failure <10%. Fig. S4. SEM images showing destructive NW transfer array on the glass substrate. Typically, yielding % failure <10%. Fig. S5. Nanowire surfaces do not significantly affect initial cell viability less than 3 hours after plating. [file 12951_2021_795_MOESM1_ESM.docx]

**Optically Transparent Vertical Silicon Nanowire Arrays for Live-Cell Imaging**

Roey Elnathan* ^a,b,c†^, Andrew W. Holle ^d,e†^, Jennifer Young ^d,e†^, Marina A. George ^b,c^ , Omri Heifler ^f,g^ , Andriy Goychuk ^h^ , Erwin Frey ^h^ , Ralf Kemkemer ^j,k^ , Joachim P. Spatz ^j,l^ , Alon Kosloff ^f,g*^ Fernando Patolsky ^f,g*^, and Nicolas H. Voelcker ^a,b,c,i*^

**Experimental section**

**Si substrate cleaning:**

Flat silicon wafers (3″/4ʺ, p-type and n-type, 1-10 Ωcm, <100>, Siltronix, France) were cleaned by a thorough rinsing in acetone, followed by isopropyl alcohol (IPA), then drying under a nitrogen jet.

**SiNW arrays fabrication:**

The cleaned substrates were spin-coated with PMMA Resist (Copolymer resist EL9, MicroChem) at 5000 rpm for 60 s, followed by baking at 180 ºC on a hot plate for 3 min. PMMA resist (Polymer resist A2, MicroChem) was coated by spinning at 2000 RPM, followed by baking at 180 ºC for 1 min on a hot plate.

An array of circles, 200 nm diameter and 1 µm pitch, was patterned on the resist layer using a Raith 150 ultrahigh-resolution e-beam lithography system (Raith GmbH, Dortmund, Germany). EBL writing was done using a beam energy of 30 keV and 20 µm aperture size. After e-beam exposure, the patterned substrate was developed in MIBK/IPA 1:3 for 1 min, followed by rinsing with IPA for 20 s, and finally drying in a N2 stream.

A nickel-dot array was deposited by e-beam evaporation of a 100 nm thick nickel layer, at a base pressure of 10−7 Torr, at a deposition rate of 1 Å/s. The remaining resist was lifted-off in an acetone solution, washed with IPA, and dried. The nickel nano-dot arrays served as mask in the following dry etching step.

Vertical NWs arrays were fabricated by applying time-multiplexed reactive ion etching in an inductively-coupled plasma deep-reactive ion-etching machine (ICP-DRIE, PlasmaTherm SLR 770). To obtain a uniform etch profile, we have implemented a Bosch-process, that consisted of alternating cycles of etching in a flow of SF6 (10 sccm, 7 s) to etch the exposed areas, and a passivation in a flow of C4F8 (60 sccm, 7 s) to protect the side walls of the etched nanostructure by depositing a fluorinated polymer. The plasma was formed with an RF power of 600 W and a forward bias of 16 W, the substrate temperature was kept at 17 ºC by cooling with a helium flow. The etching process consisted of 54 “passivation-etching” sequences, resulting in the formation of 2.4 µm long and 200 nm diameter NW structures. The residual Ni mask was chemically removed by using a nickel etchant solution.

**Anti-adhesion surface preparation of Si substrate for subsequent NW transfer:**

A 30 nm-thick Au layer was deposited over the nanostructure array by e-beam evaporation at a base pressure of 10-7 Torr, and at a rate of 0.5 Å/s. Then, a 500 nm PMMA resist (Polymer resist A4, MicroChem) layer was applied by spin coating at 2000 RPM and baking on a hot plate at 50 ºC for 1 min followed by a temperature ramp to 130 ºC for further 5 min. The residual PMMA layer that might form on the nanostructures side walls was removed by exposure to oxygen plasma (100 W, O2-200 sccm) for 1 min. In order to selectively remove the Au layer located on the top of the nanostructures and on their sidewalls, the samples were immersed in KI Au-etchant for 3 s, then rinsed with DI water, and dried carefully by a N2 stream.

**NW harvesting and imprinting (NW transfer from Si-donor to glass-acceptor substrates):**

The coated NW sample (Au/PMMA) and the glass substrate were both spin-coated with an Ormoprime-08 (Micro Resist Technology) adhesion promoter at 2000 RPM and baked at 90 ºC for 10 s. This was followed by a temperature increase to 130 ºC for baking for an additional 5 min. Subsequently, the transfer process was done by placing a small drop of Ormostamp (Micro Resist Technology) solution on the middle of the “NWs”-containing substrate. Then, the glass substrate was placed on top of the NWs sample, with its activated side facing hte Ormostamp droplet. During this stage, the Ormostamp solution spreads between the glass and the NWs forming a thin wetting layer that bonds them by capillary forces. The attached substrates were exposed to 365 nm UV radiation at a dose of 1000 W. For a final curing and hardening of the Ormostamp transparent layer the samples were baked at 130 ºC for 30 min, where the NW substrate back-surface was in contact with the hot plate. In the final stages, the samples were separated by placing a razor blade in between the samples (only at the corner, without touching the NWs array), and a small force was applied. As a result, the Ormostamp cured layer passed to the glass substrate along with the SiNWs. For an indication of a successful transfer, we observed that the bottom Au layer passed with the NWs onto the glass side (see Figure S1). Next, in order to remove the Au layer, the glass sample with the embedded NWs was dipped in Au etchant for 5 s. Following this, the upper NW area was exposed by dipping the sample in anisole solution for 5 min, removing the embedded PMMA layer. During the previously described preparation steps, the SiNW geometry has a high aspect-ratio. Thus, NW structures might fall or adhere to each other during the various coating steps. This leads to incomplete or entangled transfer. Figure S2 shows incomplete NW array transfer onto the glass substrate, while Figure S3 shows destructive NW array transfer onto the glass substrate.

**Glass substrate cleaning:**

Clear glass substrates were cleaned by a thorough rinsing in acetone, followed by IPA washing, and dried carefully by a N2 stream. Further surface cleaning and activation was applied by exposure to oxygen plasma (100 W, O2-200 sccm) for 1 min.

**Cell culture:**

MDA-MB-231 human epithelial breast cancer cells (ECACC, Salisbury, UK) were cultured in high glucose DMEM (31966, Gibco) supplemented with 10% FBS and 1% penicillin/streptomycin (100 µg/ML) (Life Technologies, Darmstadt, Germany) and grown in a 95% air/5% CO2 mixture at 37 ^o^C.

**Microscopy:**

Nanowire substrates and glass control substrates were glued (via substrate corners) to the bottom of a well in a glass-bottom 24 well plate with Picodent dental glue (Picodent, Wipperfürth, Germany) to prevent drift during live-cell microscopy. Sterilization under UV light was performed for 20 min. MDA-MB-231 cells were trypsinized and plated at a concentration of 5,000 cells/mL in wells containing either nanowire substrates or glass control substrates. Phase contrast live-cell microscopy was performed on an Axiovert 200M inverted microscope surrounded by a cell culture chamber maintaining the environment at 37 oC and 5% CO2 (Carl Zeiss, Jena, Germany). Once cells were well-spread (~3 hours), images were collected every 5 min for 24 h using an LD Plan-Neofluar 40x Corr Ph2 M27 objective.

**Data processing and statistical analysis:**

All images were normalized so that the median of the pixel values in an image is zero, and the range of pixel values is one. Image noise was reduced by using a Non-Local means algorithm implemented in Python.[1] Stain-free, single cell segmentation and tracking was performed using a convolutional neural network based on U-Net.[2,3] A small amount of 23 images was used for the training of the neural network. To that end, all cells in a given training image were manually segmented and used as ground truth. Training was performed for 100 epochs, with 500 steps per epoch and a batch size of 1; a larger batch size was not possible due to the large dimensions of individual images (1388 x 1040 pixels, padded to 1392 x 1040 pixels). To prevent the neural network from overfitting at such a small sample size of ground truth images, artificial data augmentation was performed in the form of affine image transformations (rotation, scale and translation), mirroring, as well as smoothed random transformations of the images.[4]

Cell segmentation was performed with the neural network connection weights obtained from the training procedure. Specifically, given the neural network weights $w_{i}$ after the $i$-th training epoch, the neural network performed a nonlinear mapping from the phase contrast image $I(x,y)$ to a smooth predicted mask $M_{i}\left( x,y \right)=p\left( I,w_{i} \right)$. Each value of the smooth predicted mask lies in the interval $M_{i}\left( x,y \right)\in\left[ 0\ldots1 \right].$ Here, we interpreted $M_{i}(x,y)$ as a “confidence value” of cellular material being present at the coordinates $(x,y)$. Using the network weights of the last 10 training iterations that improved the prediction (i.e. reduced the loss function) of the neural network, we proposed 10 different smooth segmentation masks $M_{i}\left( x,y \right)$ for each phase contrast image $I(x,y)$. Finally, we generated an average of the 10 different smooth segmentation masks: $M\left( x,y \right)=\left\langle M_{i}\left( x,y \right) \right\rangle_{i}$. We used a “confidence region” of $M\left( x,y \right)\geq0.999$ to determine an “inner mask”, which we empirically found to give a good separation of adjacent cells. Then, using this “inner mask” as seed, we performed a watershed segmentation of $M\left( x,y \right)$ within a “confidence region” of $M\left( x,y \right)\geq0.25$. The automatically segmented cell masks were cleaned by hand to delete false positive segmentation results. The resulting data were then analyzed in Python and Microsoft Excel to determine dynamic values for cell area, cell aspect ratio, and cell position, which were used to calculate cell spreading rate and cell velocity. Individual time-dependent cell centroid positions were linked into cell tracks by using the Python package Trackpy[3]. Two-tailed Student’s t tests were used to compare cellular data obtained on nanowires vs. glass substrates.

**Additional data**


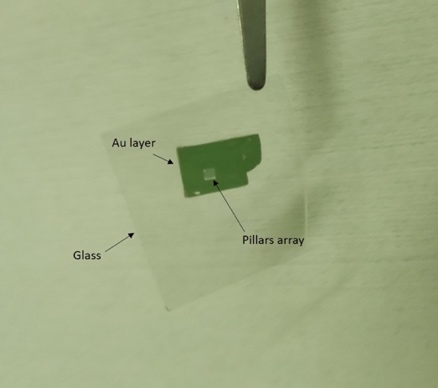


**Fig. S1:** Glass substrate following the application of Ormostamp layer and separation from the Si substrate. The transferred Si-NW array with its bottom Au coating is indicated by the arrows***.***


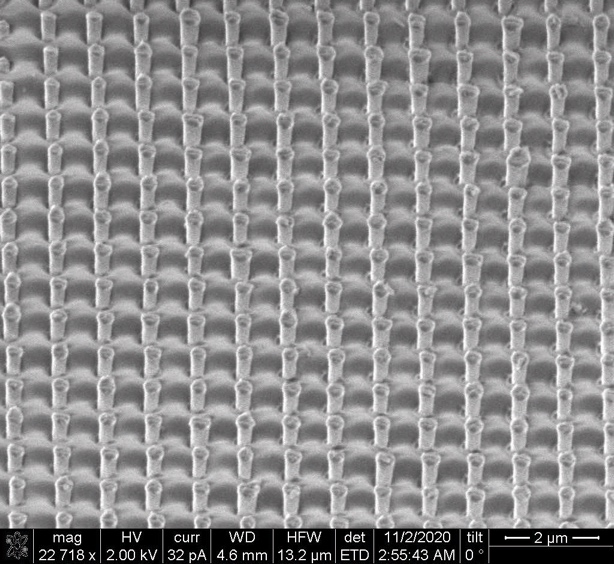

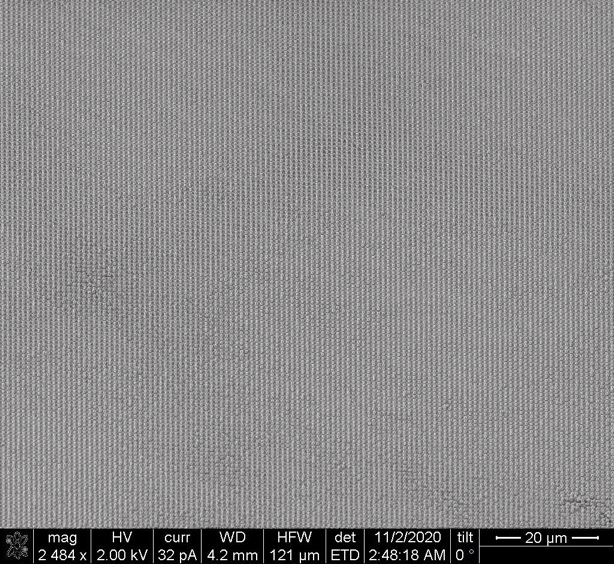


(b)

(a)

**Fig. S*2*:** Zoom-out and zoom-in SEM images showing an additional imprinted NW geometry:

height 1 µm and 0.8 µm spacing.


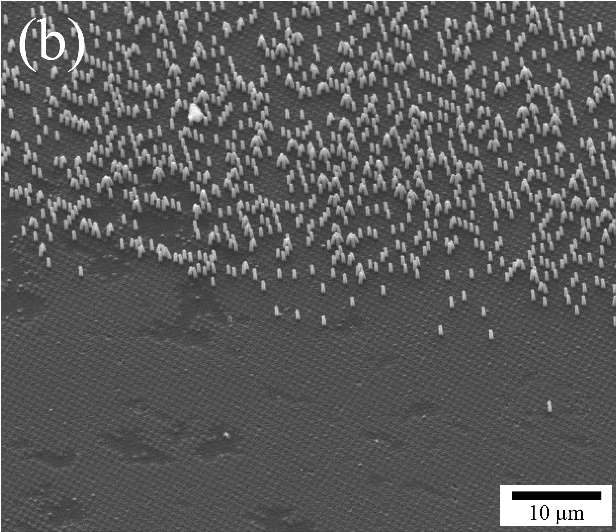

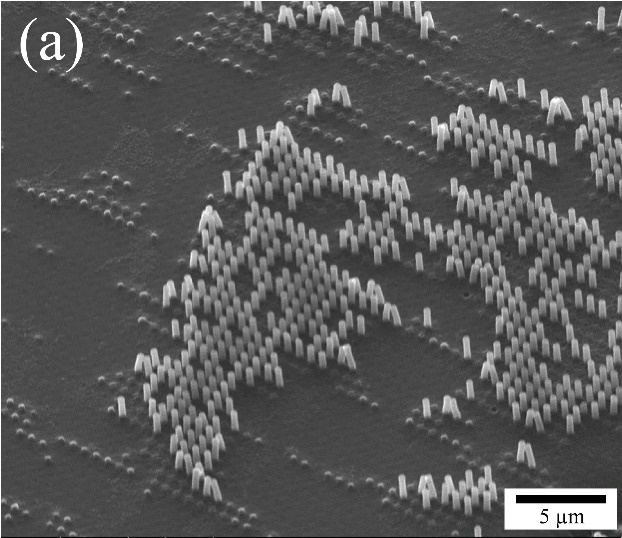


**Fig. S3:** SEM images showing incomplete NW transfer array onto the glass substrate. Typically, yielding % failure <10%.


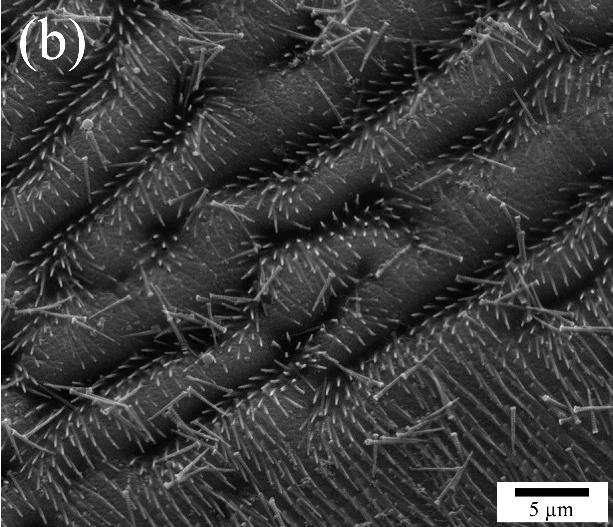

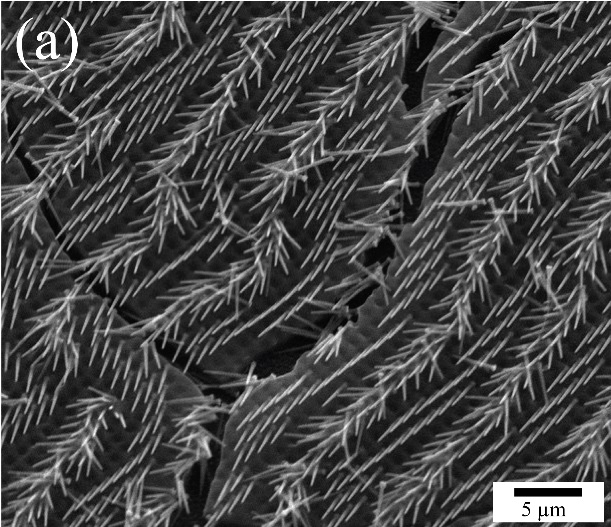


**Fig. S4:** SEM images showing destructive NW transfer array on the glass substrate.

Typically, yielding % failure <10%.

**Fig. S5:** Nanowire surfaces do not significantly affect initial cell viability less than 3 hours after plating.
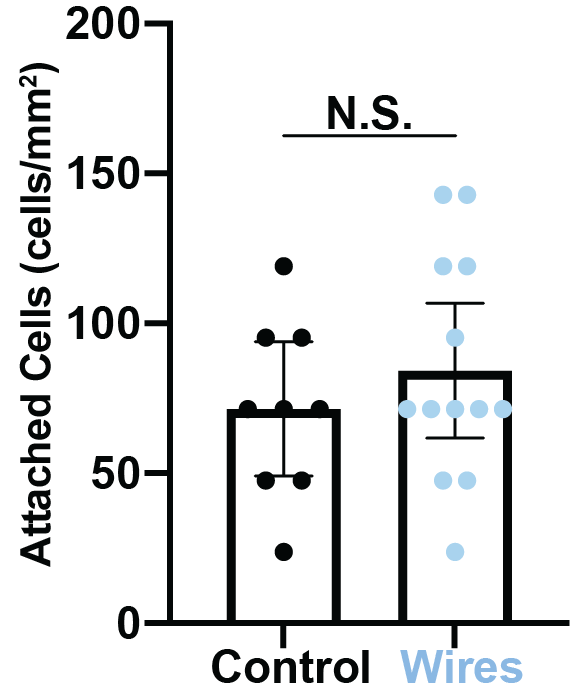


**Video 1**. Phase contrast video of MDA-MB-231 cells on a flat glass substrate.

**Video 2**. Phase contrast video of MDA-MB-231 cells on a transparent VA-SiNW substrate.

**Video 3**. Video of MDA-MB-231 cells with overlaid automatic cell segmentation results (p (x,y)) and cell contours at p (x,y) = 0.25. The glyph 'x' denotes the respective centroid, with the current object ID.

All videos are 215 *×* 162 µm.

**References:**

1. J. Darbon, A. Cunha, T. F. Chan, S. Osher and G. J. Jensen, "*Fast nonlocal filtering applied to electron cryomicroscopy*" 2008 5th IEEE International Symposium on Biomedical Imaging: From Nano to Macro, Paris, 2008, pp. 1331-1334, doi: 10.1109/ISBI.2008.4541250.
2. T. Falk, Mai, D., R. Bensch, U-Net: deep learning for cell counting, detection, and morphometry. *Nat Methods* 16, 67–70 (2019). https://doi.org/10.1038/s41592-018-0261-2
3. Olaf Ronneberger, Philipp Fischer, and Thomas Brox ‘U-Net: Convolutional Networks for Biomedical Image Segmentation’ <https://arxiv.org/pdf/1505.04597.pdf>
4. P. Y. Simard, D. Steinkraus and J. C. Platt, "*Best practices for convolutional neural networks applied to visual document analysis*" Seventh International Conference on Document Analysis and Recognition, 2003. Proceedings., Edinburgh, UK, 2003, pp. 958-963, doi: 10.1109/ICDAR.2003.1227801
5. https://zenodo.org/record/3492186#.X255H5MzZTZ
